# Supplementary material for: A human model of Batten disease shows role of CLN3 in phagocytosis at the photoreceptor–RPE interface
Source: Commun Biol. 2021 Feb 5;4:161. doi: 10.1038/s42003-021-01682-5 (PMC7864947; doi:10.1038/s42003-021-01682-5)
Supplement: Supplementary file 2 — Supplementary Information [file 42003_2021_1682_MOESM2_ESM.pdf]

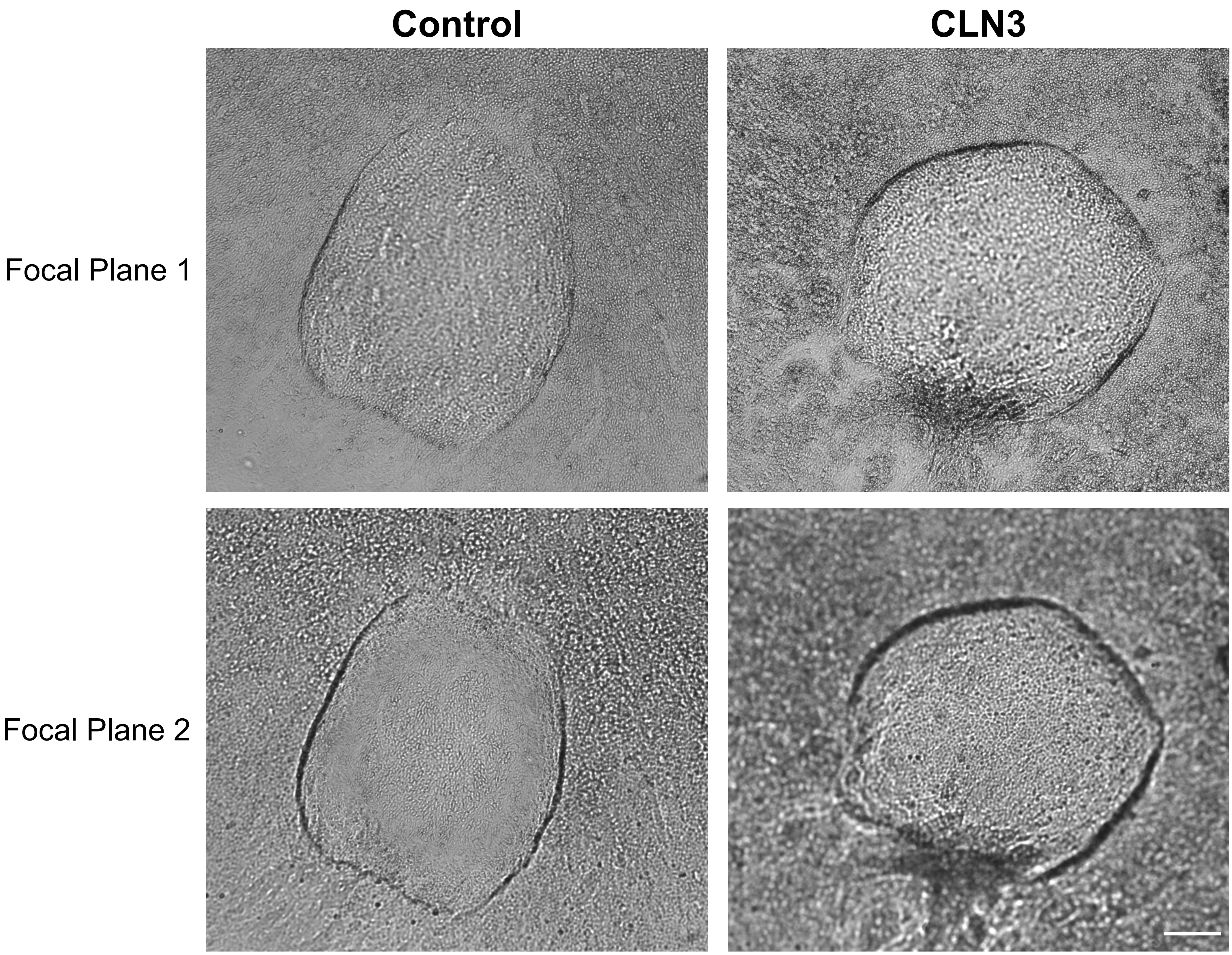

Supplementary Figure 1. Presence of fluid domes in control and CLN3 disease hiPSC-RPE cultures. *Representative light microscopy images of control and CLN3 disease hiPSC-RPE cells at two different focal planes showing the presence of fluid domes consistent with the formation of a polarized RPE monolayer. Of note, images in focal plane 1 (top panel) focuses on hiPSC-RPE monolayer within the fluid dome and images in focal plane 2 (bottom panel) focuses on hiPSC-RPE monolayer outside the fluid dome. (n ≥ 3). Scale bar = 50 µm.*

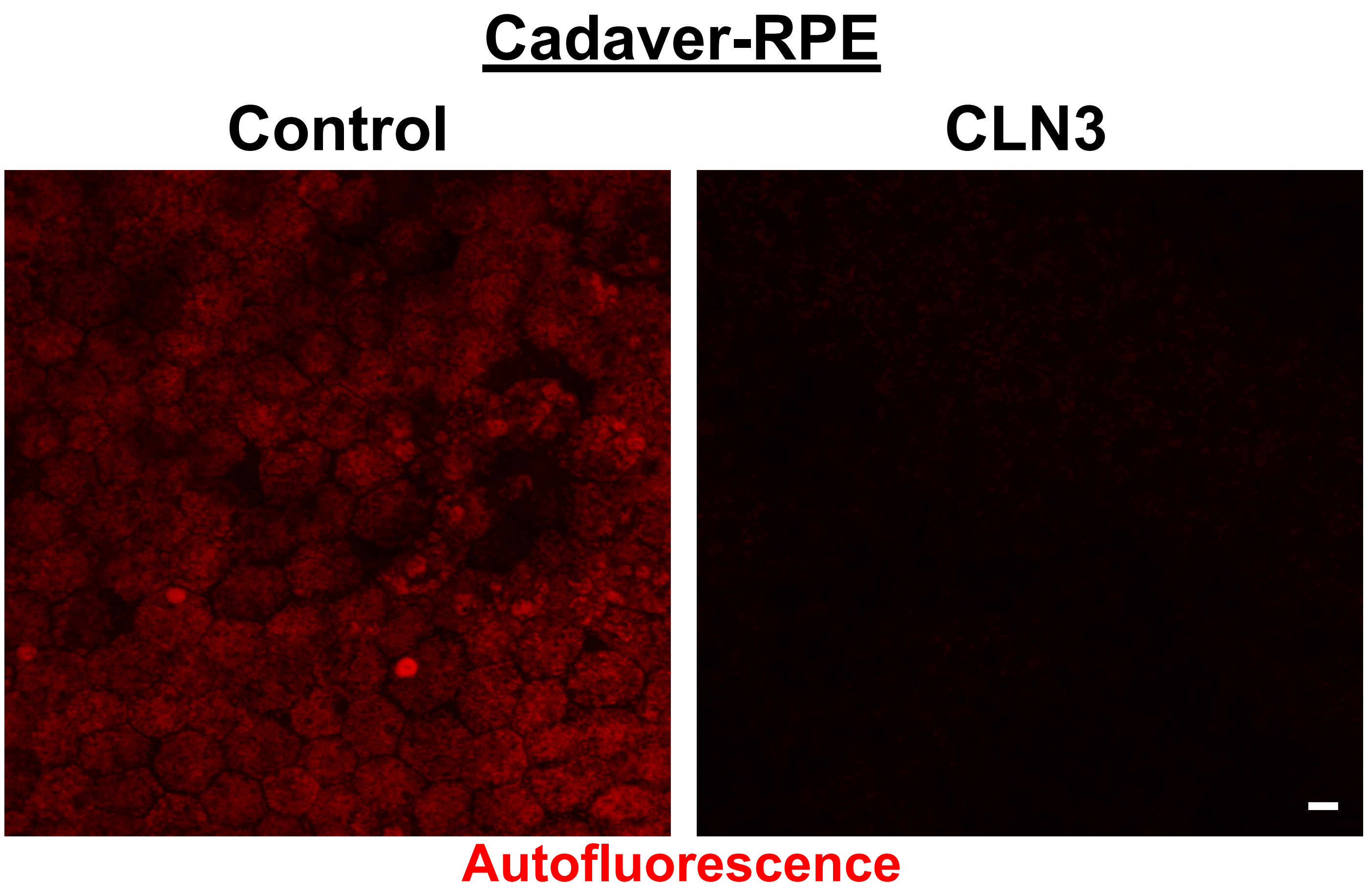

Supplementary Figure 2. Reduced lipofuscin accumulation in CLN3 disease donor RPE monolayer. *Representative confocal microscopy images showing decreased autofluorescent material accumulation in spectral wavelength consistent with that of POS-digestion product (lipofuscin) (ex: 546 nm, em: 560-615 nm) in CLN3 disease donor RPE cells compared to RPE cells acquired from an adult healthy donor eye. Scale bar = 10 µm.*

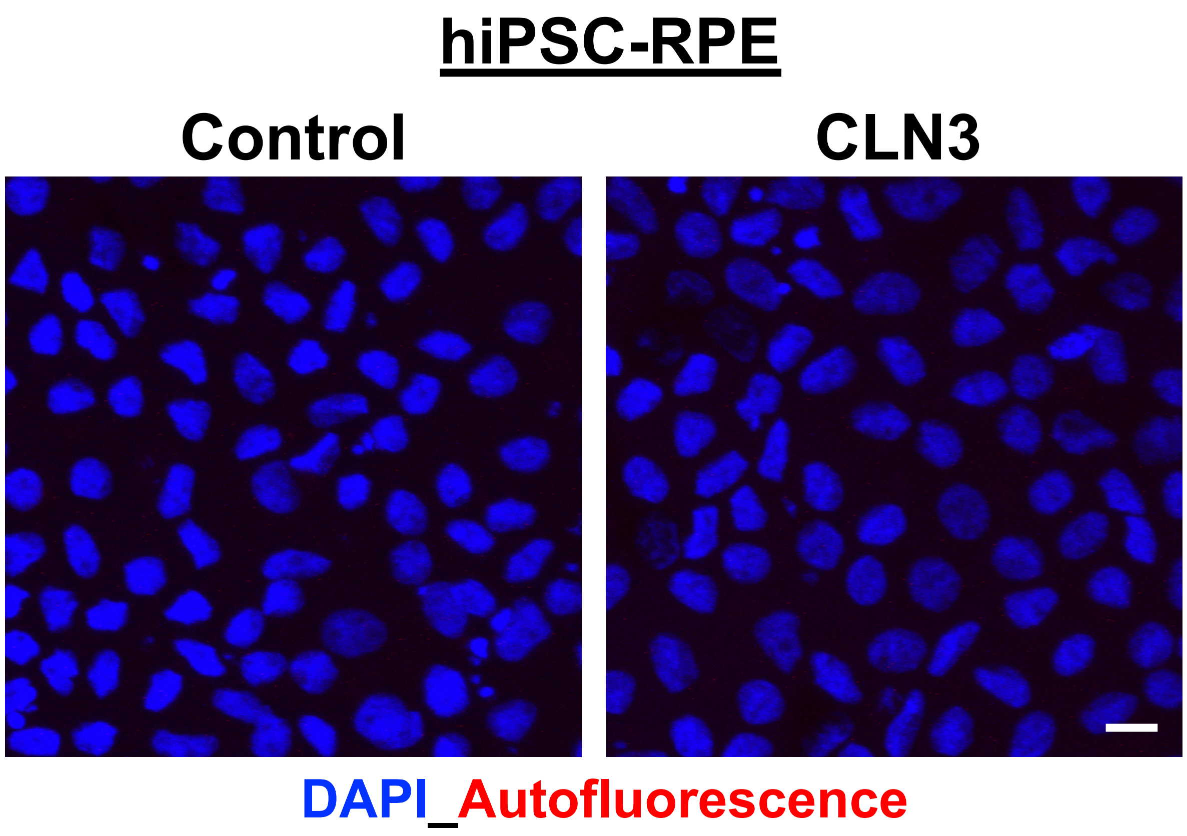

Supplementary Figure 3. Control and CLN3 disease hiPSC-RPE cells possess no autofluorescence in the absence of POS feeding. *Representative confocal microscopy images showing no baseline autofluorescence (ex: 546 nm, em: 560-615 nm) in control and CLN3 disease hiPSC-RPE cells in the absence of POS feeding (scale bar = 10 µm) (n ≥ 3).*

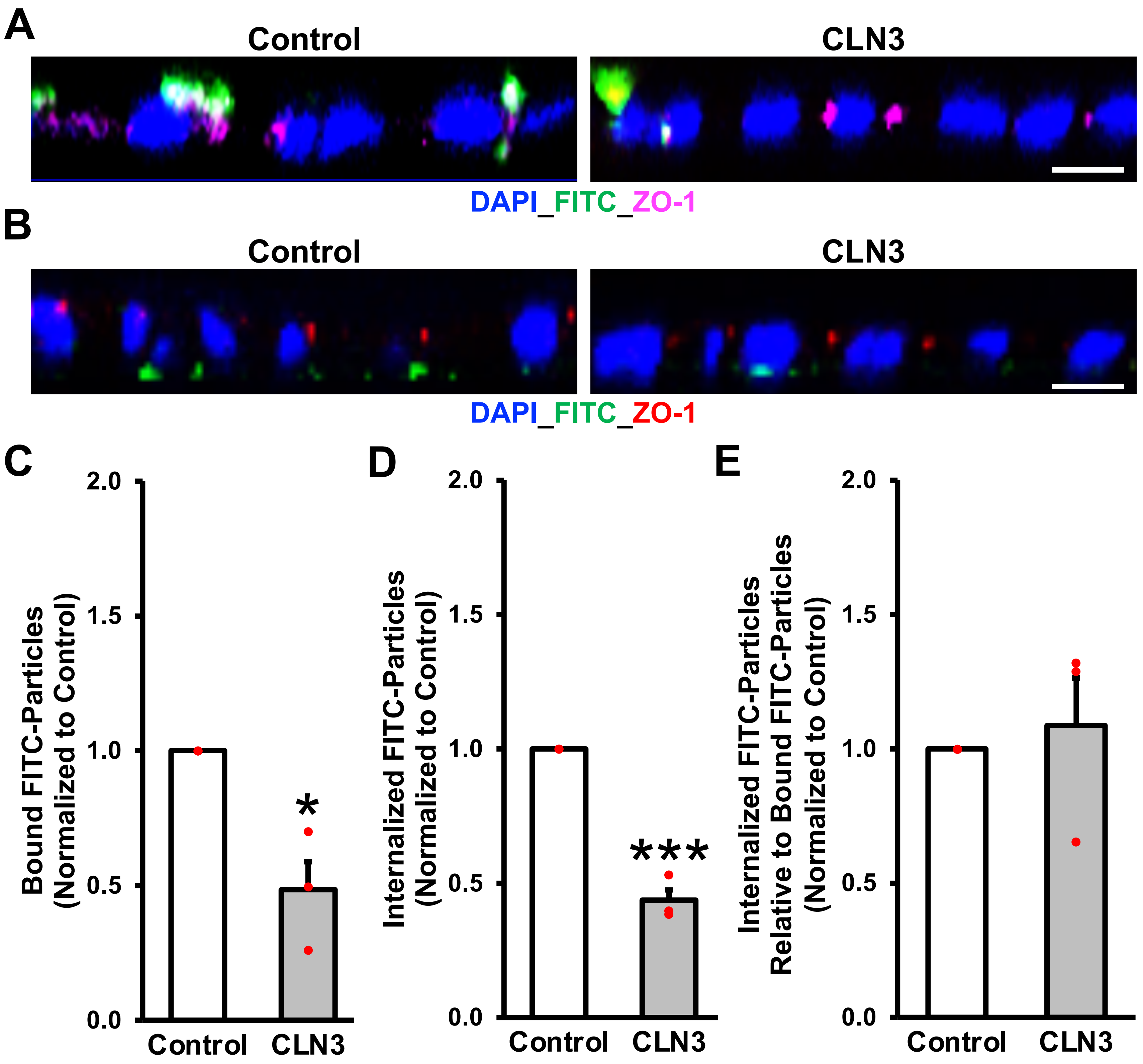

Supplementary Figure 4. Decreased POS binding leads to reduced POS internalization by CLN3 disease hiPSC-RPE cells. A, B*) Representative orthogonal view of FITC-POS fed (2h at 37°C) RPE monolayers following immunocytochemical and confocal microscopy analyses showing decreased number of FITC-POS particles both apical (consistent with reduced FITC-POS binding (****A****) and basal (consistent with reduced FITC-POS internalization) (****B****) of tight junction marker, ZO-1, in CLN3 disease hiPSC-RPE cells compared to control hiPSC-RPE cells (scale bar = 10 µm) (n=3).* ***C, D****) Quantitative analyses using a published protocol [46] that uses the position of FITC-POS-relative ZO-1 to evaluate levels of bound and internalized POS showed reduced number of both bound (above ZO-1) (****C,*** *p = 0.015) and internalized (below ZO-1) (****D,*** *p = 0.00027) FITC-POS particles in parallel cultures of CLN3 disease hiPSC-RPE compared to control hiPSC-RPE cells post 2h FITC-POS feeding (n=3).* ***E****) Consistent with a POS binding defect in CLN3 disease hiPSC-RPE cells, normalization of internalized FITC-POS to bound FITC-POS in control and CLN3 disease hiPSC-RPE showed no difference in the number of internalized FITC-POS particles in control vs. CLN3 disease hiPSC-RPE cells (n=3, p = 0.71). Two-tailed unpaired Student's t-test performed for all statistical analysis. *p<0.05, ***p<0.0005.*

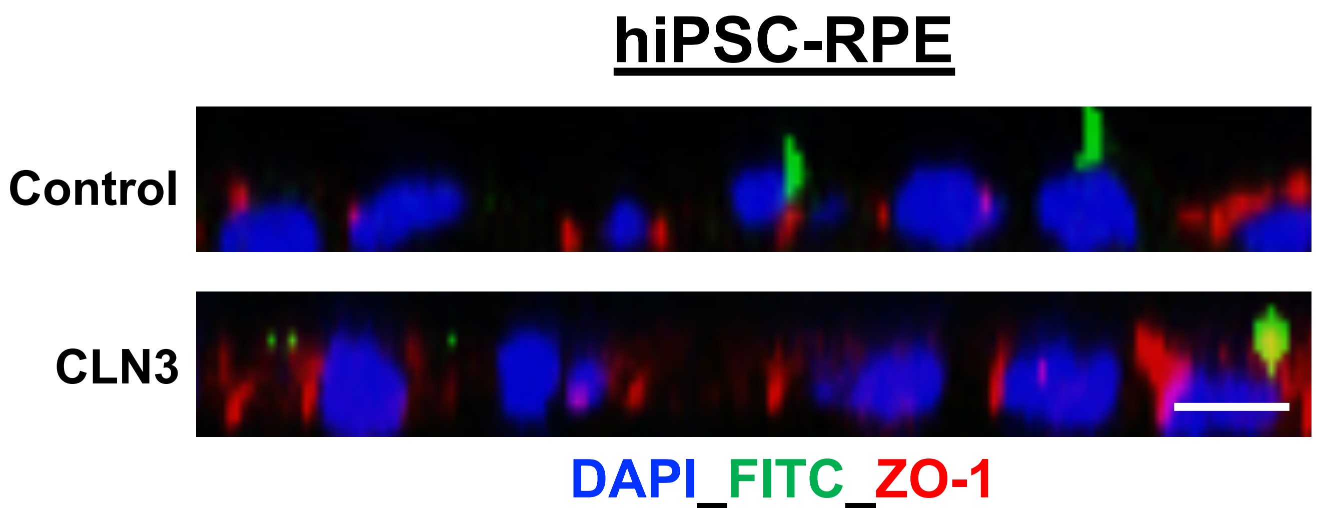

Supplementary Figure 5. FITC-POS is predominantly apical of ZO-1 in hiPSC-RPE cells fed POS at 17ºC. *Consistent with published studies showing efficient binding but limited internalization of POS at 17ºC [47], representative confocal images post FITC-POS feeding for 30 min at 17ºC and immunocytochemical analyses with ZO-1 antibody showing apically localized FITC-POS particles relative to ZO-1 in orthogonal view of both control and CLN3 disease hiPSC-RPE cultures (scale bar = 10 μm). Note: Cell nuclei are stained with DAPI. (n =4).*

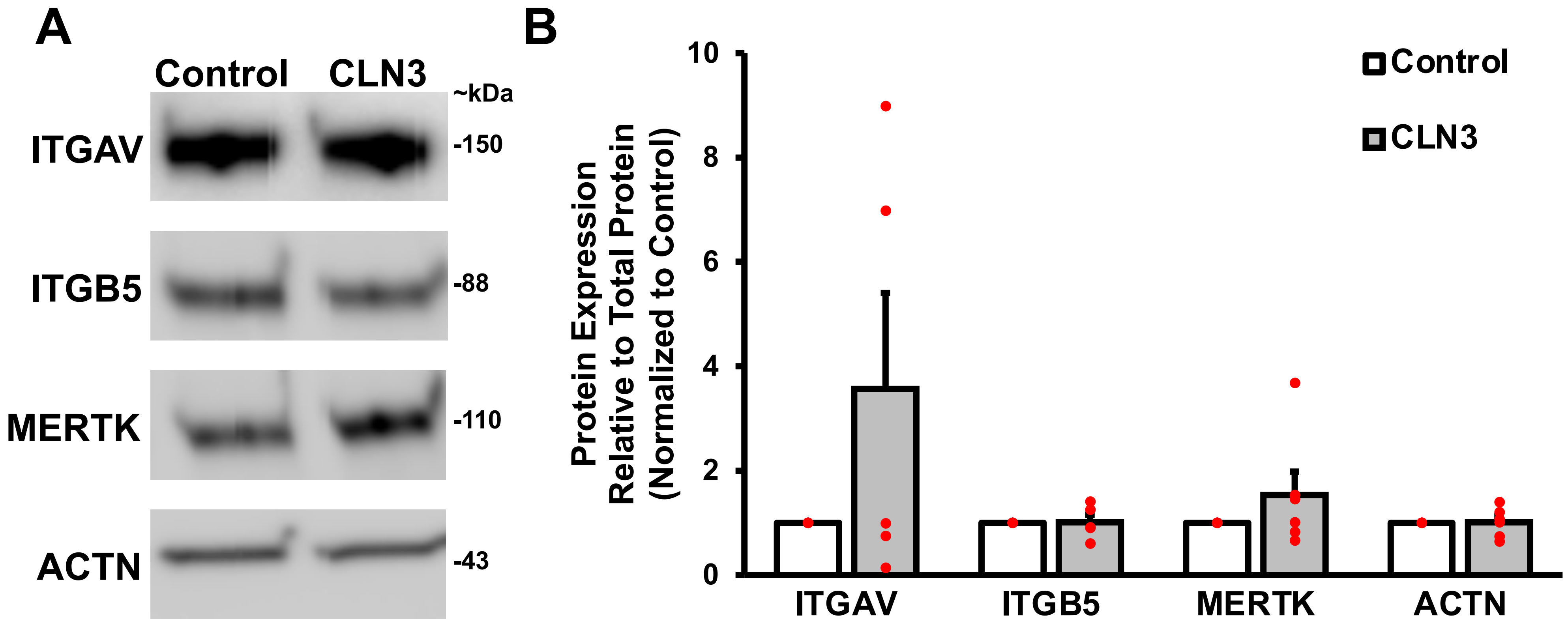

Supplementary Figure 6. Similar protein levels of RPE binding and engulfment receptor in control vs. CLN3 disease hiPSC-RPE cells. A, B*)* *Representative Western blot images (**A**) and quantitative Western blot analyses (**B**) showing similar protein levels of RPE binding receptor, integrin alpha 5 (ITGAV) (n=5, p = 0.20), integrin beta 5 (ITGB5) (n=5, p = 0.93), and RPE engulfment receptor, MER proto-oncogene, Tyrosine Kinase (MERTK) (n=6, p = 0.27), relative to total protein levels in control vs. CLN3 disease hiPSC-RPE. Similarly, no difference in levels of ACTN relative to total protein levels were seen between control and CLN3 disease hiPSC-RPE (n=6, p = 0.94). Two-tailed unpaired Student's t-test performed for all statistical analysis.*

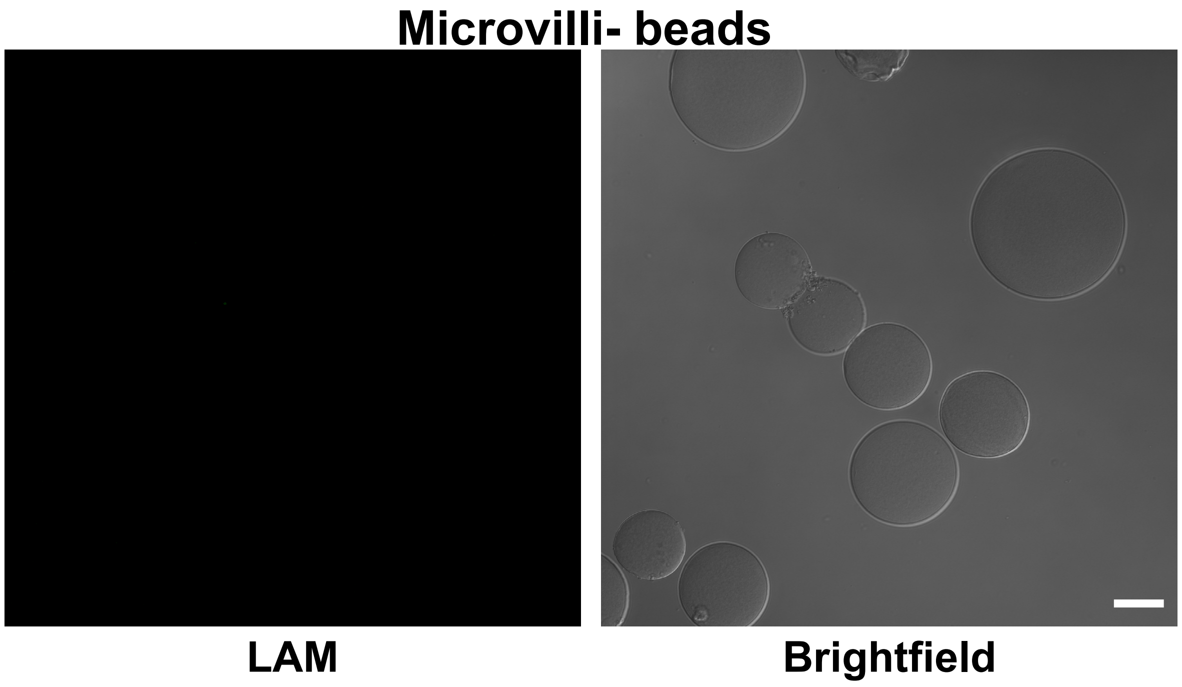

Supplementary Figure 7. LAM is not present in the control hiPSC-RPE microvilli-bound lectin-agarose beads. *Consistent with a prior publication [50], representative confocal microscopy images showing lack of RPE-extracellular matrix protein, Laminin (LAM), in microvilli-adhering lectin-agarose beads collected from control hiPSC-RPE cultures post execution of the microvilli isolation protocol (scale bar = 10 μm) (n ≥ 3).*

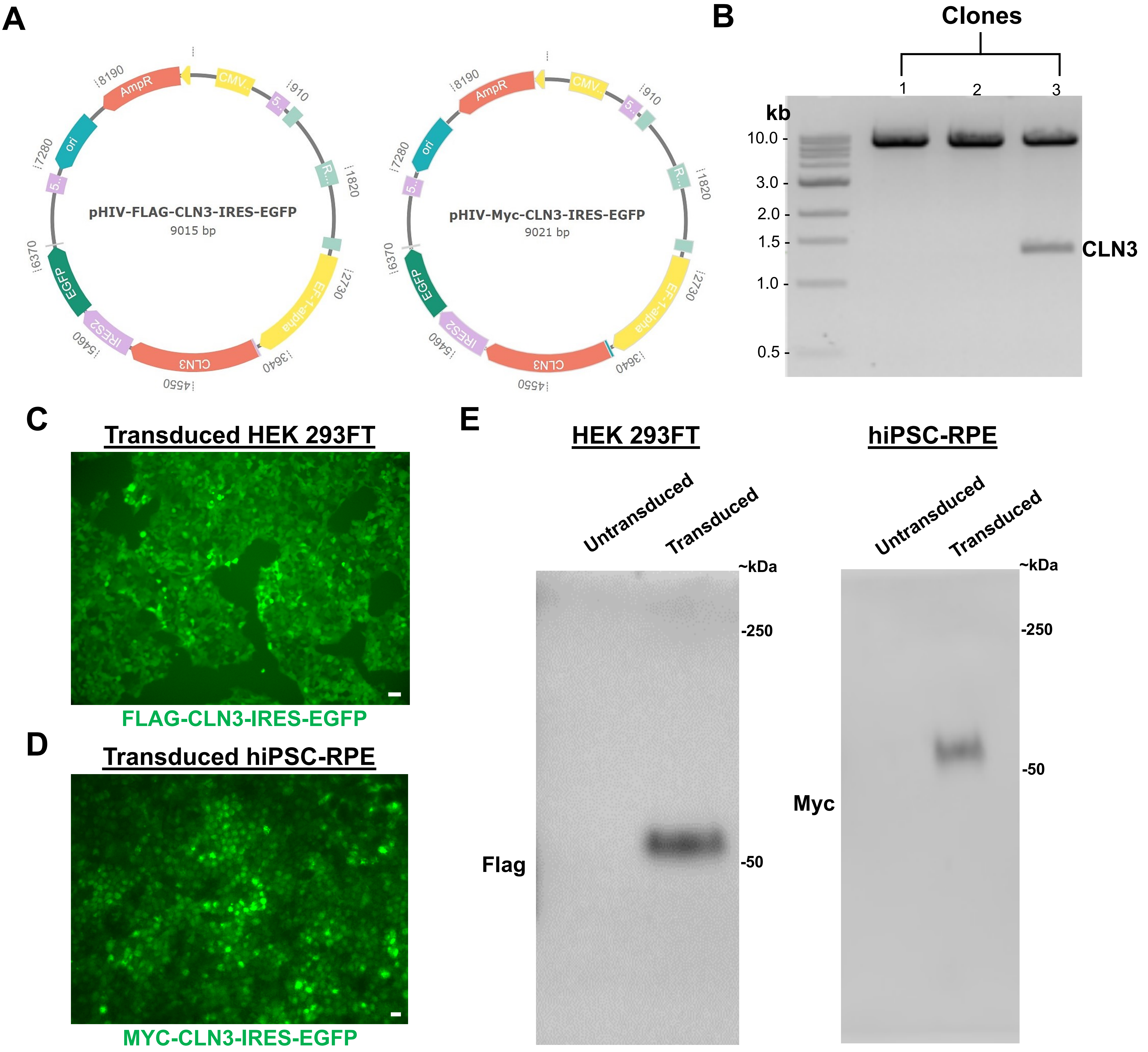

***Supplementary Figure 8. Generation and over-expression of FLAG and MYC epitope tagged CLN3 lentiviral vector in HEK293FT and control hiPSC-RPE cells. A**) Schematic vector map of FLAG-CLN3 and MYC-CLN3 used in this study. Specific features of the vector including EF-1α: the eukaryotic elongation factor-1 alpha promoter and N-terminus FLAG-tagged CLN3 and N-terminus MYC-tagged CLN3 are demarcated. **B**) Gel electrophoresis subsequent to restriction enzyme digestion using HpaI and BamHI endonucleases, shows the expected FLAG-CLN3 restriction fragment in clone 3. **C, D**) Representative fluorescent microscopy images showing HEK 293FT cells (top panel, **C**) and control hiPSC-RPE cells (bottom panel, **D**) stably transduced with lentivirus encoding FLAG-CLN3 (top panel) and MYC-CLN3 (bottom panel). Notably, a large proportion of cells expressing enhanced green fluorescent protein (EGFP) from a downstream IRES element can be clearly visualized in both top and bottom panel(scale bar = 20 µm). **E**) Representative Western blot images showing the expression of FLAG-CLN3 fusion protein in the transduced HEK 293FT cells (probed with an anti-FLAG antibody) and MYC-CLN3 (probed with an anti-MYC antibody in transduced control hiPSC-RPE cells but no expression of FLAG and MYC in untransduced HEK 293FT and control hiPSC-RPE cells, respectively.*

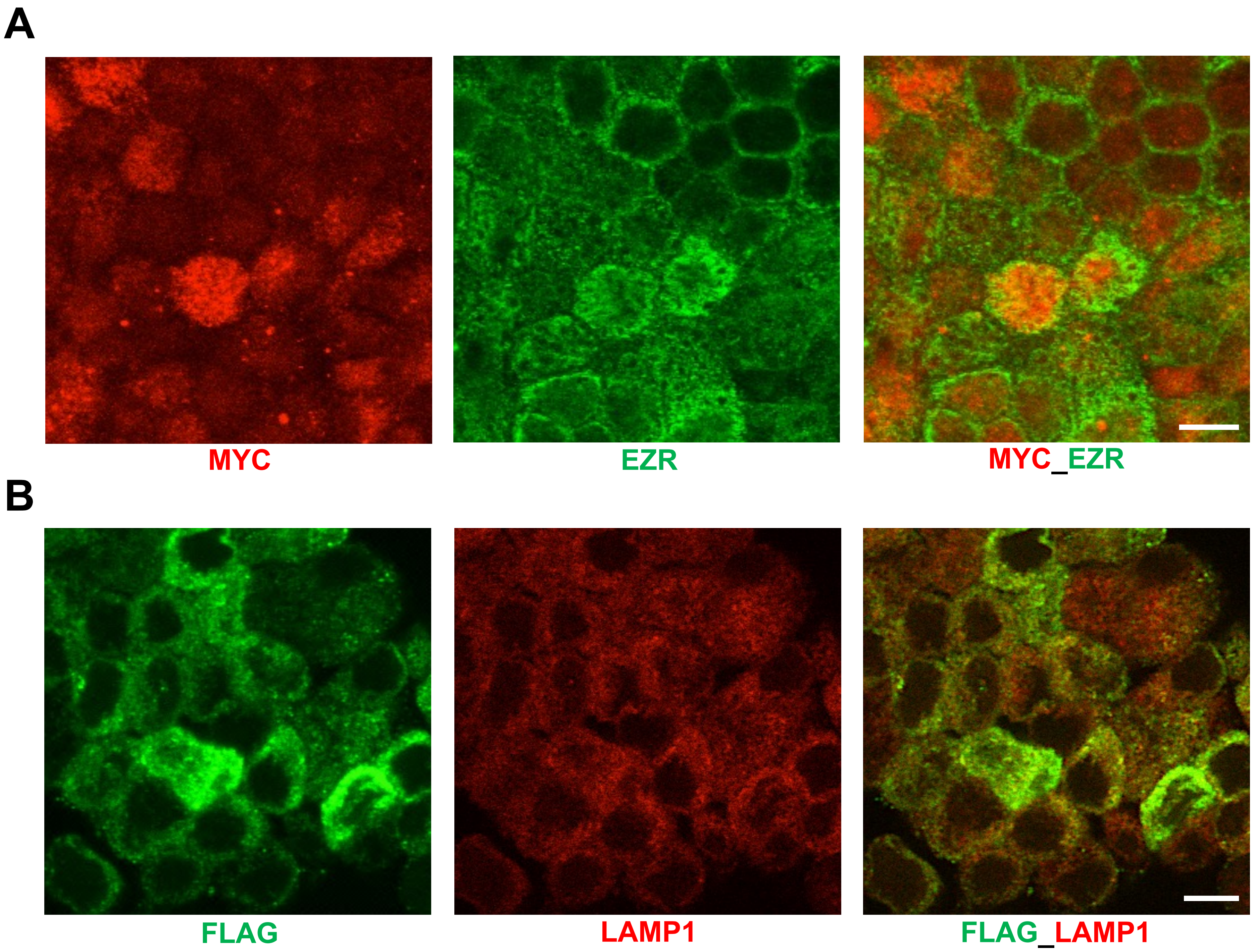

***Supplementary Figure 9. A proportion of overexpressed CLN3 protein is present in the microvilli and lysosome of hiPSC-RPE cells. A**) Representative confocal microscopy images post immunocytochemical analyses with MYC and EZR antibodies showing co-localization of MYC-CLN3 and RPE microvilli protein, EZR in CLN3 disease hiPSC-RPE cells transduced with pHIV-MYC-CLN3-IRES-EGFP lentiviral vector. **B**) Representative confocal microscopy images post immunocytochemical analyses with FLAG and LAMP1 antibodies showing co-localization of FLAG-CLN3 with LAMP1, in control hiPSC-RPE cells transduced with pHIV-FLAG-CLN3-IRES-EGFP lentiviral vector (scale bar = 10 μm) (n ≥ 3).*

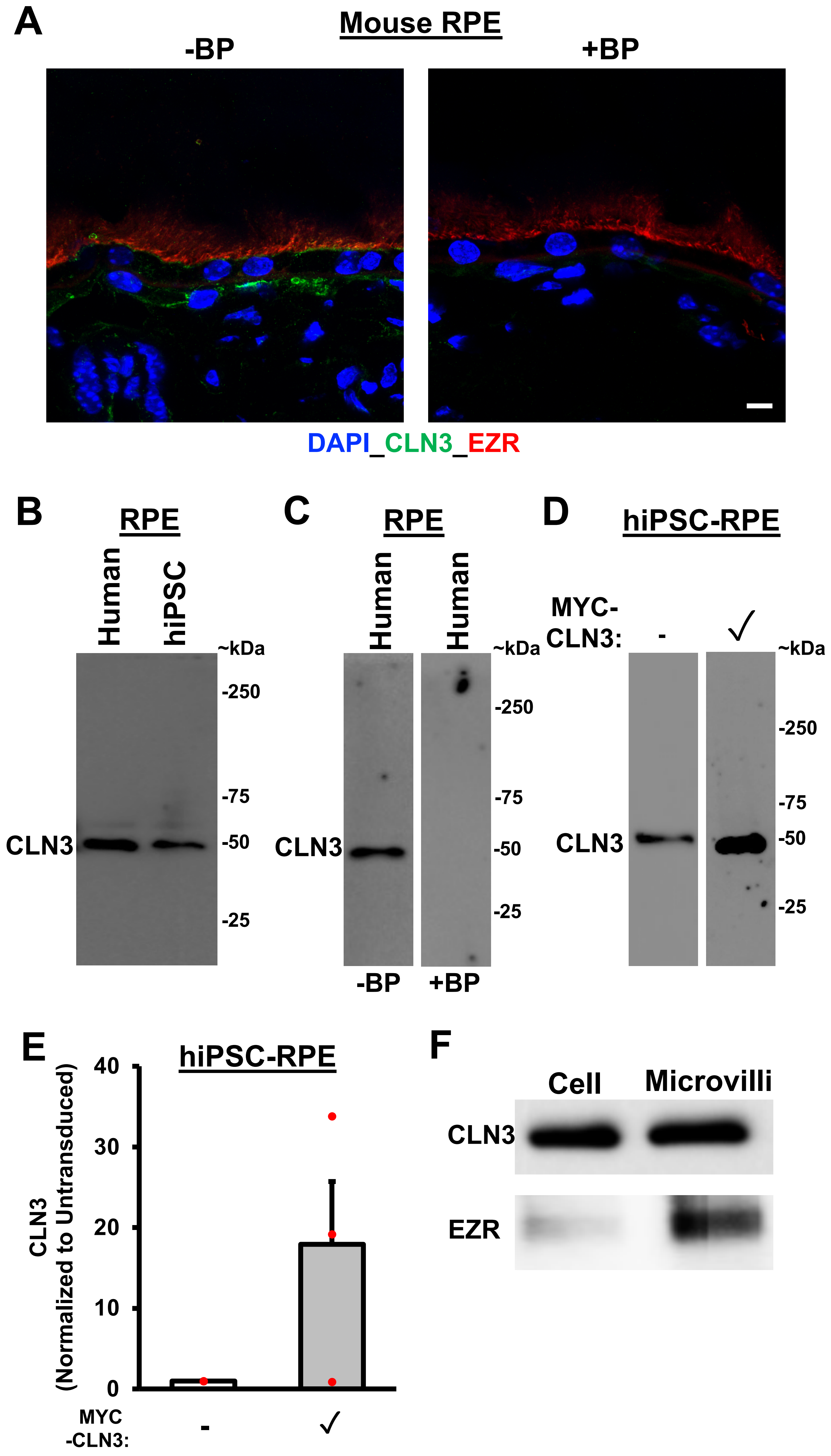

Supplementary Figure 10. Validation of CLN3 antibody in primary, hiPSC and mouse RPE samples. A*)* *Representative confocal microscopy images after immunostaining with CLN3 and ezrin (EZR) antibodies showing strong presence of endogenous CLN3 and co-localization of endogenous CLN3 and RPE microvilli protein, EZR, in wild-type (WT) mouse RPE (left panel). Notably, CLN3 blocking peptide abolishes detection of endogenous CLN3 but not EZR in mouse RPE sections (scale bar =10μm). (n ≥ 3).****B****) Representative Western blot image showing two bands of endogenous CLN3 at ~ 50 kDa (consistent with CLN3 glycosylation) in primary human RPE and control hiPSC-RPE samples (~20 μg total protein loaded on SDS-PAGE for each sample) analyzed with CLN3 antibody.* ***C****) Representative Western blot images showing a strong band ~50 kDa in primary human RPE samples (~15 μg total protein loaded on SDS-PAGE) (left panel) analyzed with CLN3 antibody. Furthermore, neutralization of CLN3- antibody with CLN3 blocking peptide abolishes the ~50kda band in the same Western blot (right panel).* ***D, E****) Representative Western blot images (****D****) and quantitative analyses (****E****) showing ~6.5-fold increase in levels of CLN3 protein relative to the total protein in the MYC-CLN3 transduced control hiPSC-RPE cells compared to untransduced control hiPSC-RPE cells (untransduced n=4, transduced n=3; p = 0.087).* ***F****) Representative western blot images of control hiPSC-RPE cell vs. microvilli fraction, post RPE apical microvilli isolation using lectin-agarose beads [50, 94], showing the presence of endogenous CLN3 in both the cell pellet and microvilli fraction. In contrast, and as expected, the RPE microvilli protein, EZR, is predominantly present in the RPE microvilli fraction (n=3). Two-tailed unpaired Student's t-test performed for all statistical analysis.*

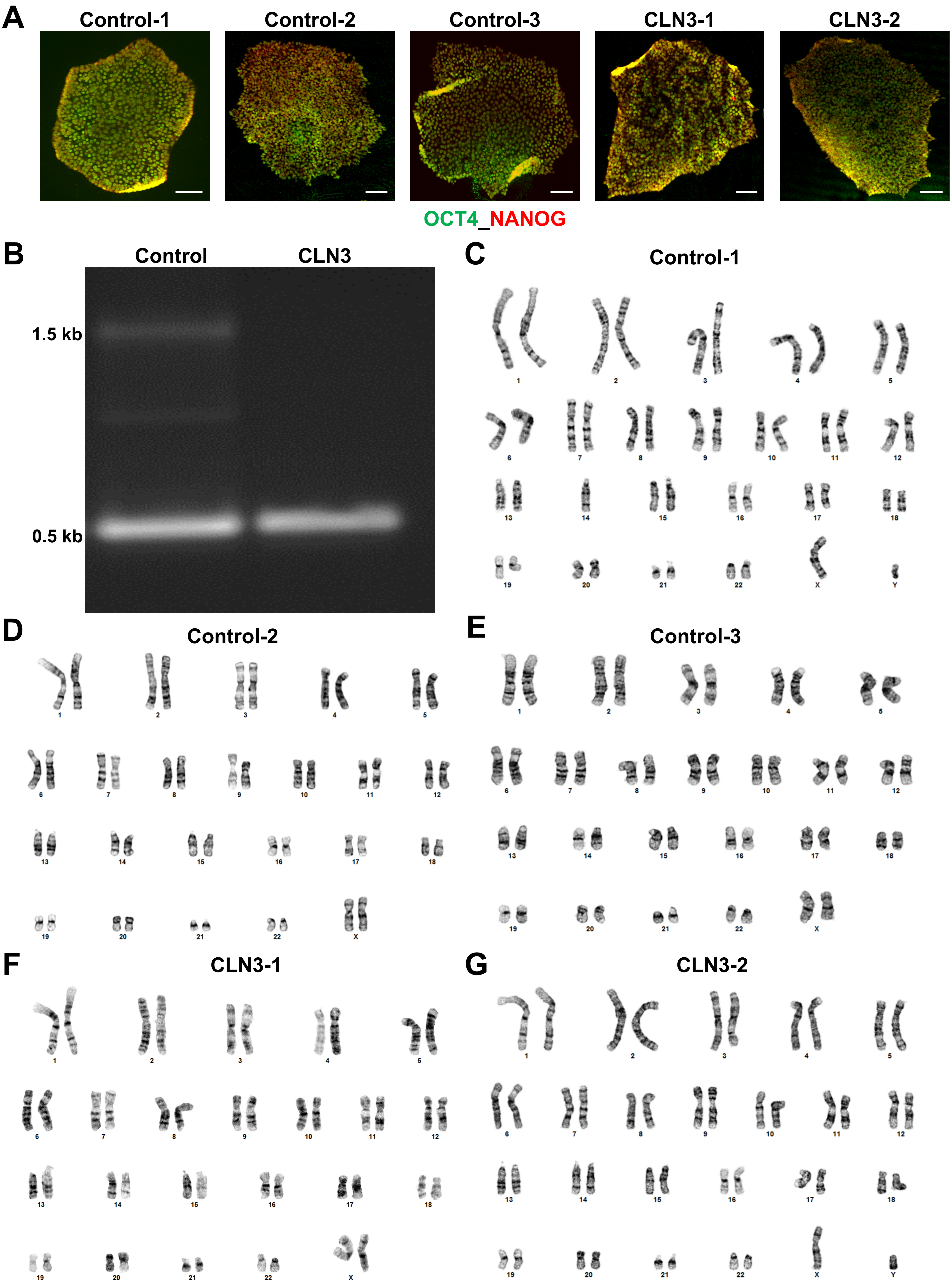

***Supplementary Figure 11. Characterization of control and CLN3 disease hiPSC-RPE cells. A**) Representative confocal microscopy images showing robust expression of pluripotency markers, OCT4 and NANOG, in the 3 distinct control and two different CLN3 disease hiPSC lines with the common 966 bp deletion used in this study (scale bar = 10 µm). **B**) Representative gel image of PCR product resolved by electrophoresis (run using previously published primers [92]) confirming the 966 bp deletion spanning exon 7 and 8 in CLN3 disease hiPSCs but not control hiPSCs. **C-G**) Standard karyotyping images showing the intact chromosomal integrity in 3 distinct control and 2 distinct CLN3 disease (harboring the 966 bp deletion) hiPSC lines used in this study.*

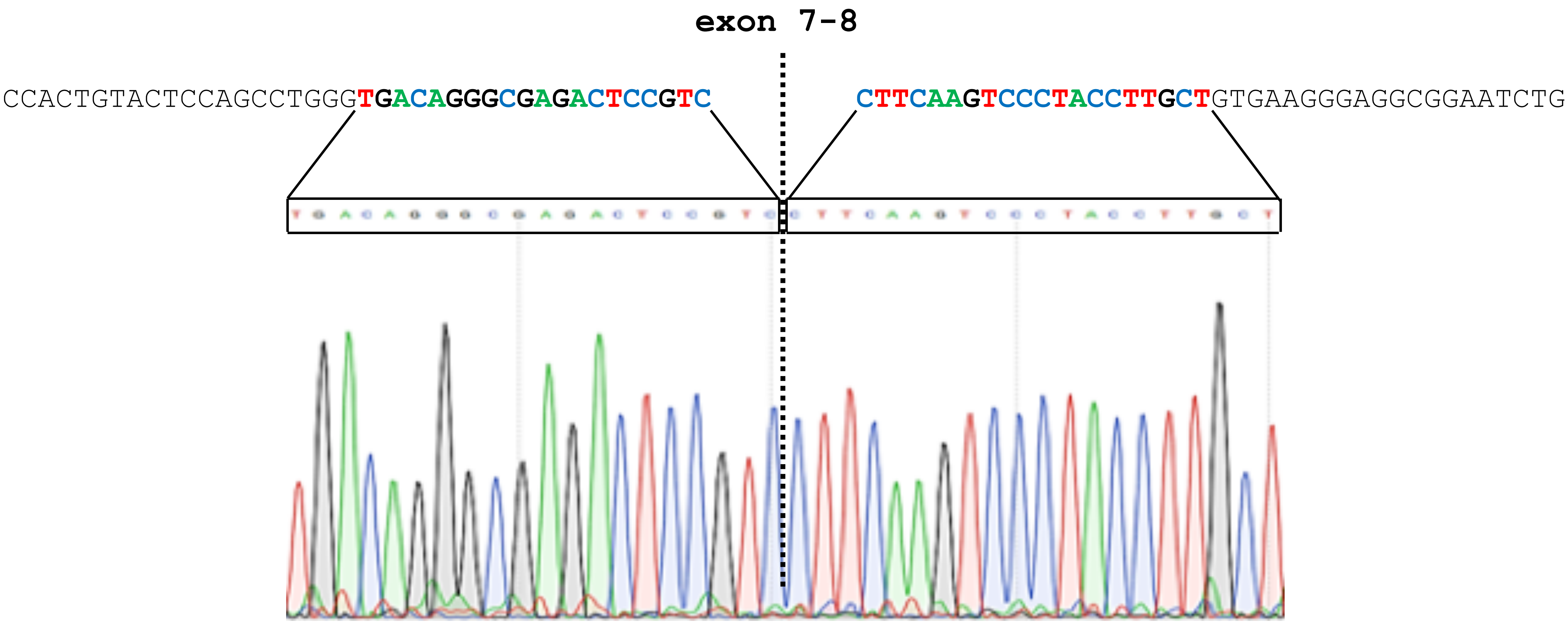

Supplementary Figure 12. Confirmation of 966 bp gene deletion spanning exon 7 and 8 in CLN3 disease hiPSCs. *Representative sequencing chromatogram showing the presence of 966 bp deletion spanning exon 7 and 8 in CLN3 disease hiPSCs. Of note sequencing confirmed the expected 966 bp deletion in both the CLN3 disease patient hiPSC lines.*

A Raw Western blot data for Figure 1E

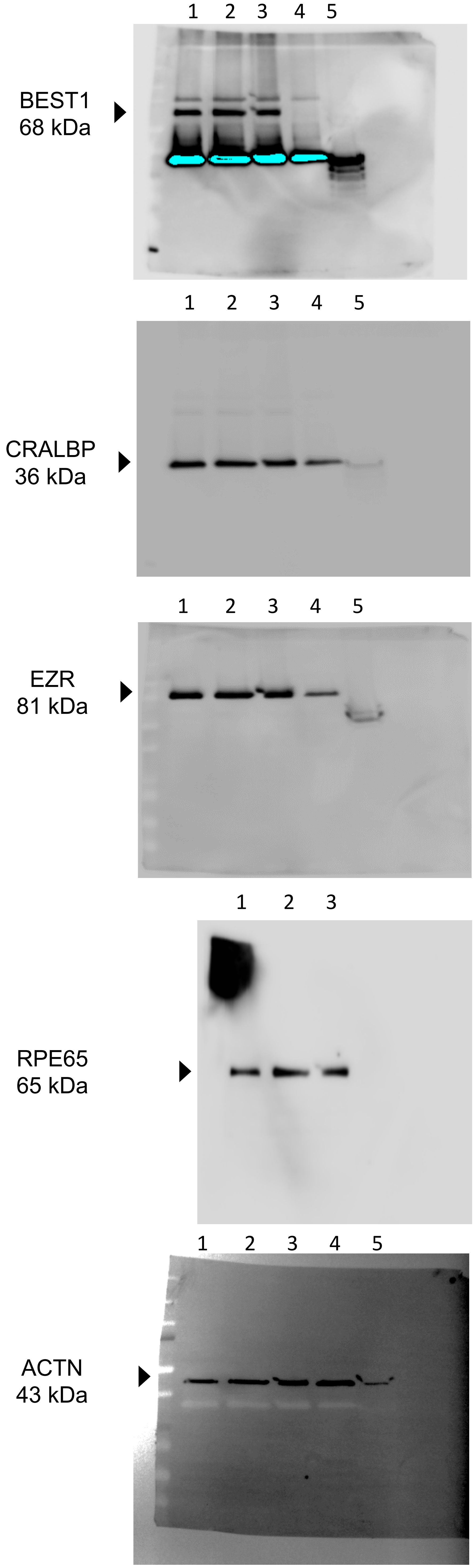

Lane 1 and 2 on all blots/ probe of equivalent amount of the same protein samples in lane 1 and 2 shown in Figure 1E

B Raw Western blot data for Figure 4B

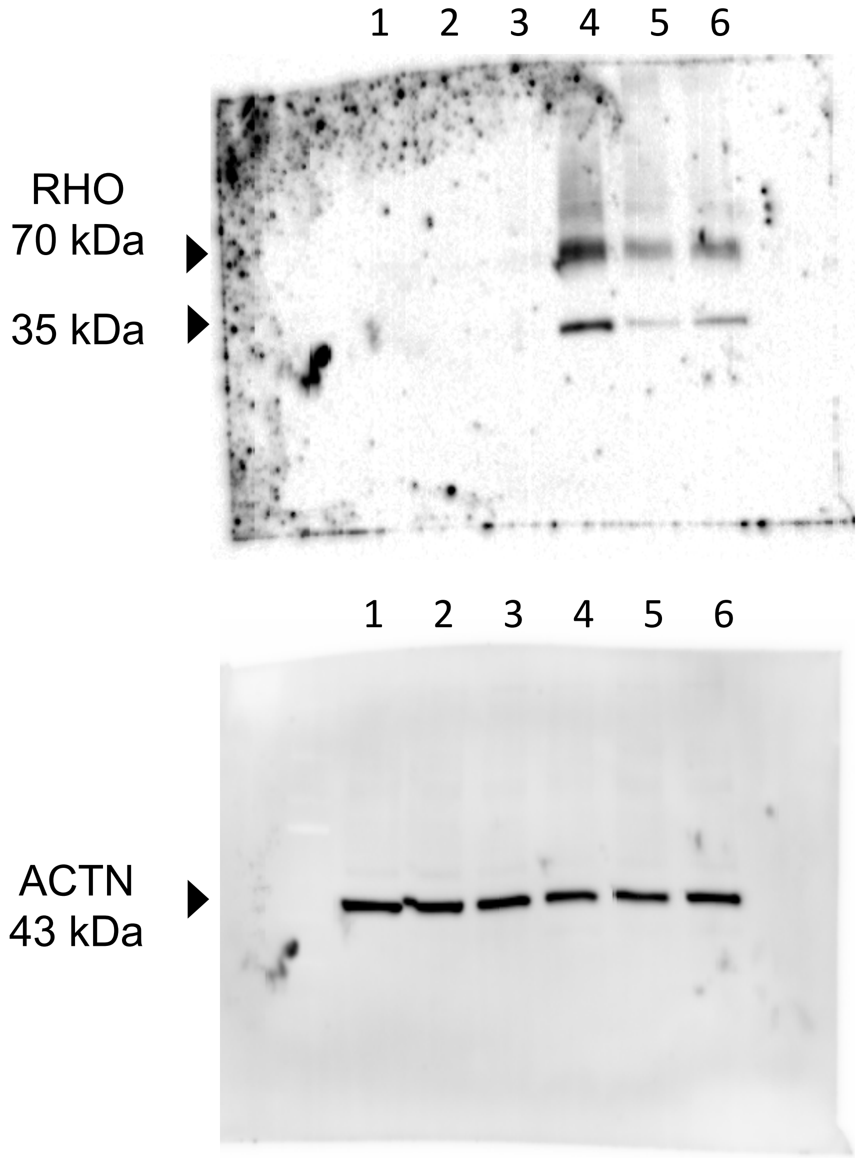

Lane 4 and 5 on all blots/ probe of equivalent amount of the same protein samples in lane 4 and 5 shown in Figure 4B

C Raw Western blot data for Figure 4E

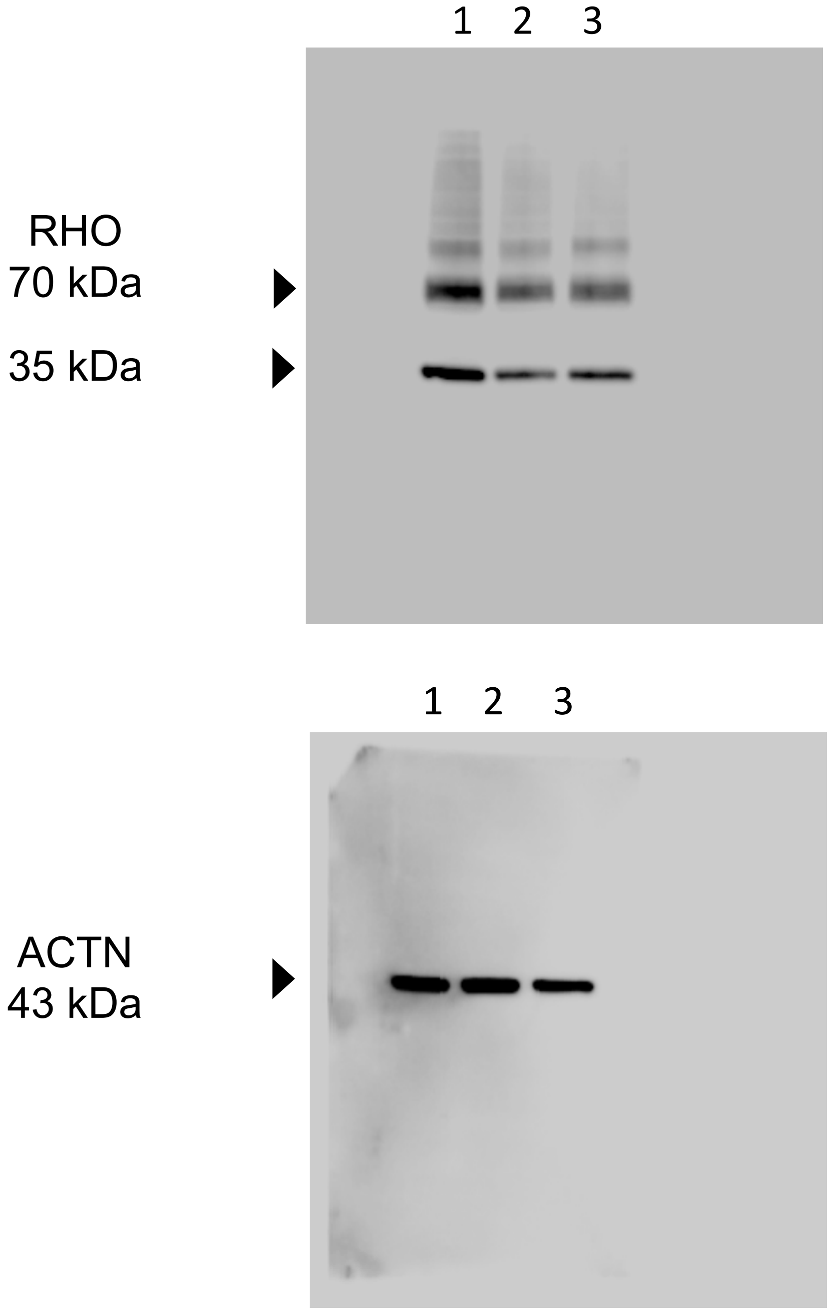

Lane 1 and 2 on all blots/ probe of equivalent amount of the same protein samples in lane 1 and 2 shown in Figure 4E

D Raw Western blot data for Figure 7G

1 2 3

CLN3
~50 kDa

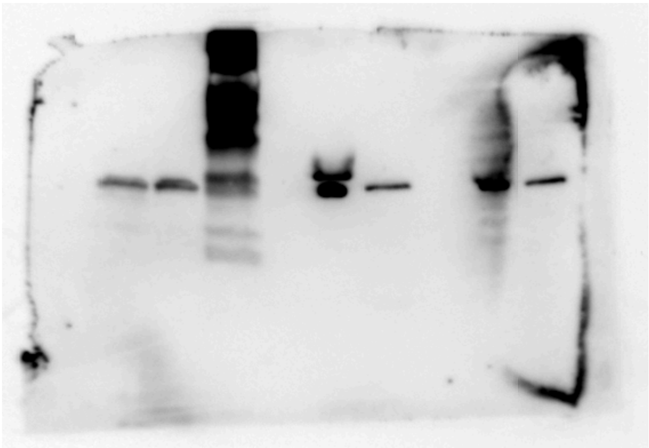

Lane 1, 2, and 3/ probe of equivalent amount of the same protein samples in lane 1, 2, and 3 shown in Figure 7G

E Raw Western blot data for Figure 8E

1 2 3 4

RHO
70 kDa

35 kDa

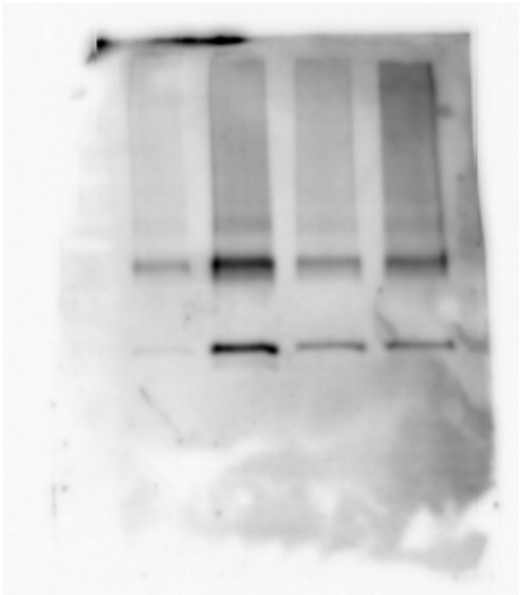

1 2 3 4

ACTN
43 kDa

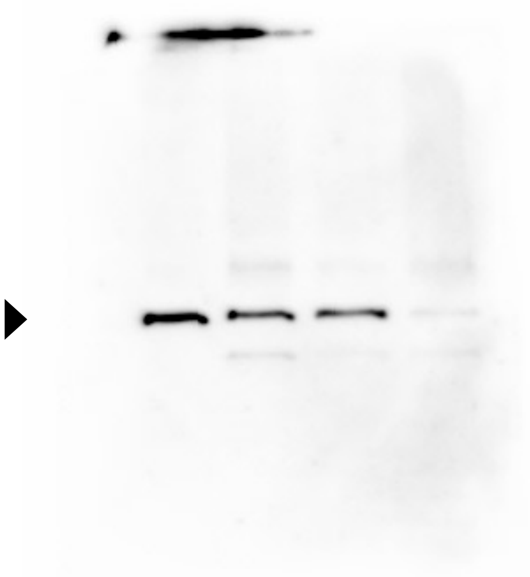

Lane 1 and 2 on all blots/ probe of equivalent amount of the same protein samples in lane 1 and 2 shown in Figure 8E

F Raw Western blot data for Figure 8F

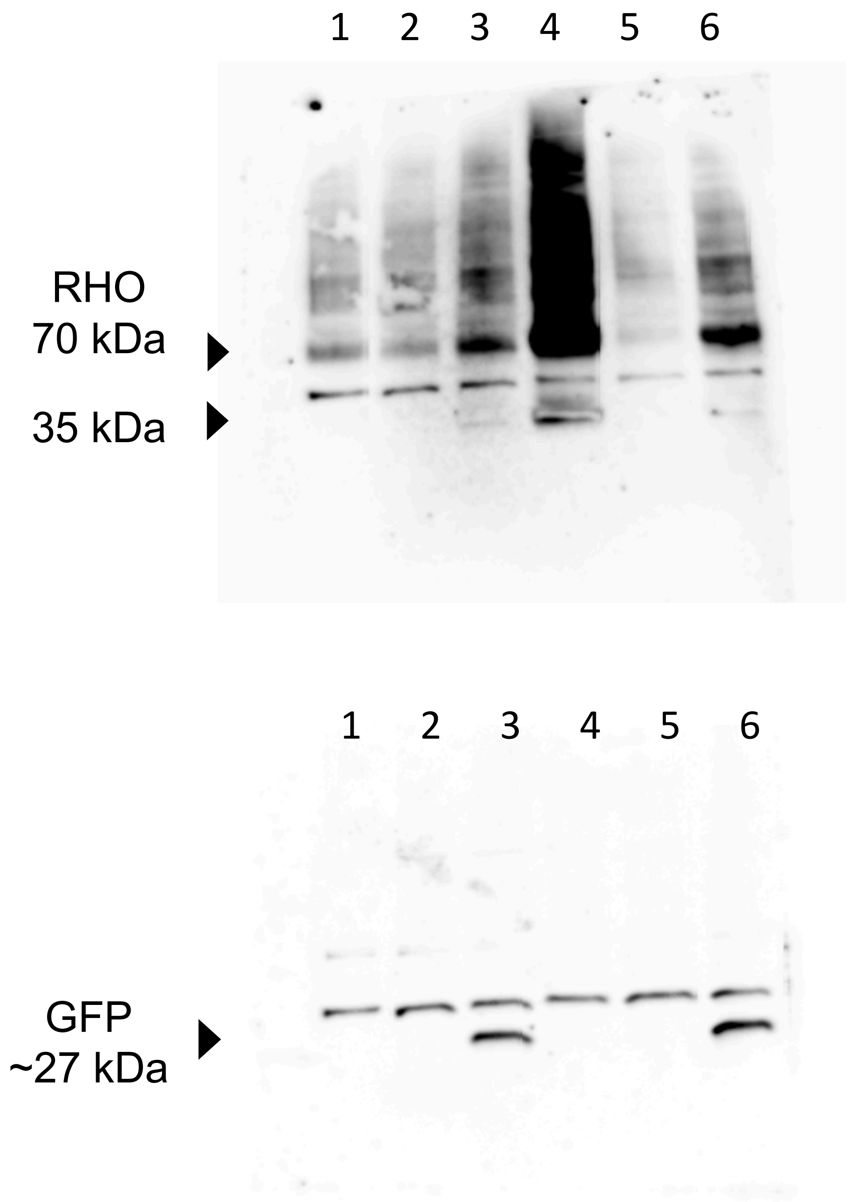

Lane 5 and 6 on all blots/ probe of equivalent amount of the same protein samples in lane 5 and 6 shown in Figure 8F

G Raw Western blot data for Figure S6

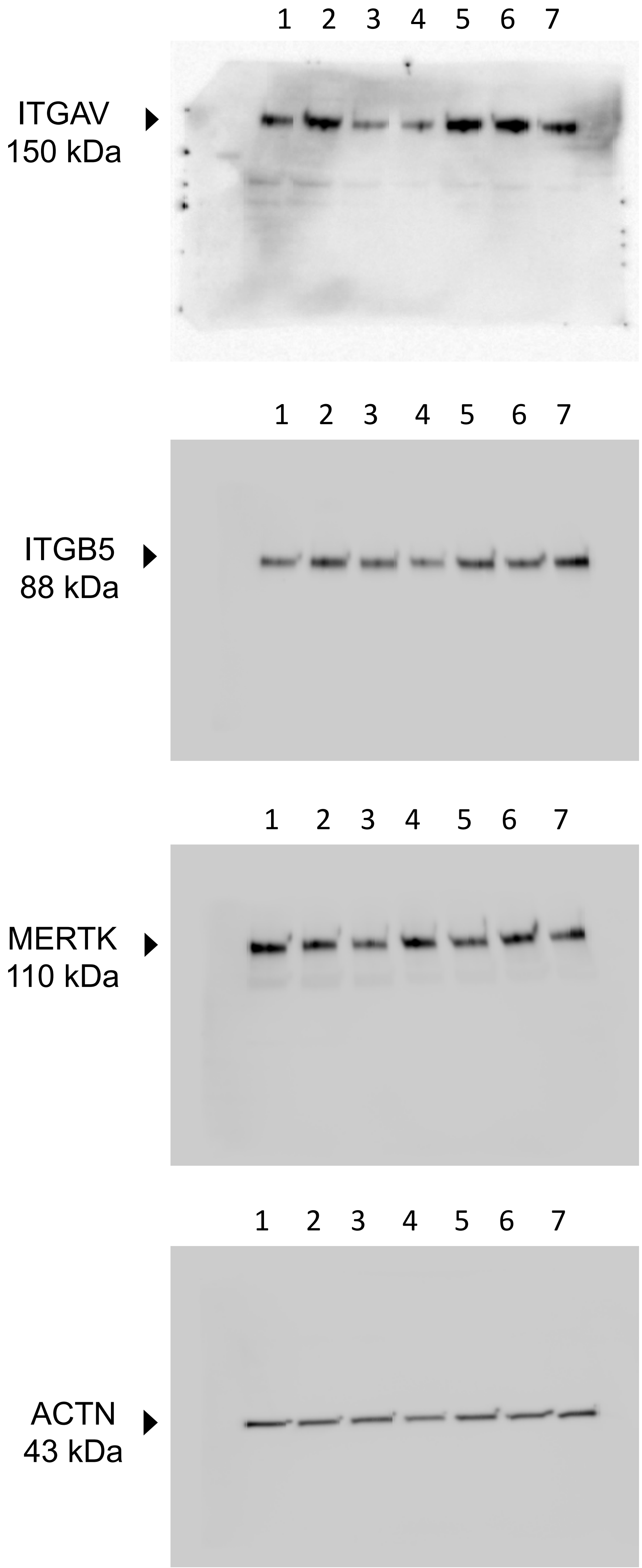

Lane 5 and 6 on all blots/ probe of equivalent amount of the same protein samples in lane 5 and 6 shown in Figure S6A

H Raw Western blot data for Figure S8

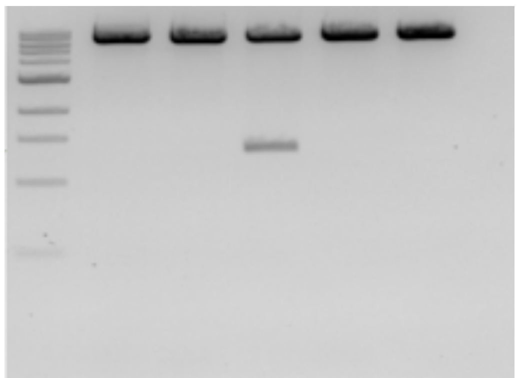

Lane 1, 2, 3, and 4/ probe of equivalent amount of the same protein samples in lane 1, 2, 3, and 4 shown in Figure S8B

I Raw Western blot data for Figure S8

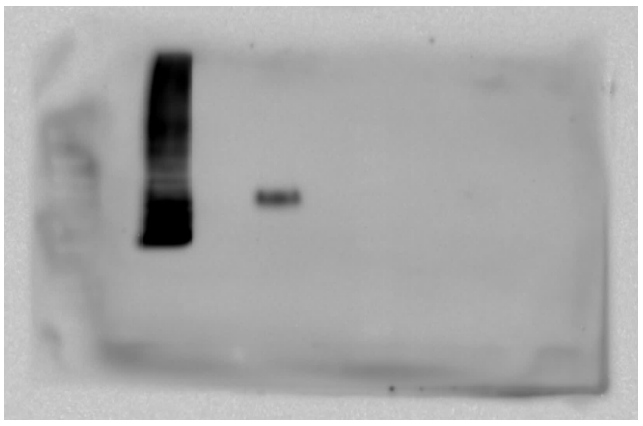

Lane 3 and 4/ probe of equivalent amount of the same protein samples in lane 3 and 4 shown in Figure S8E

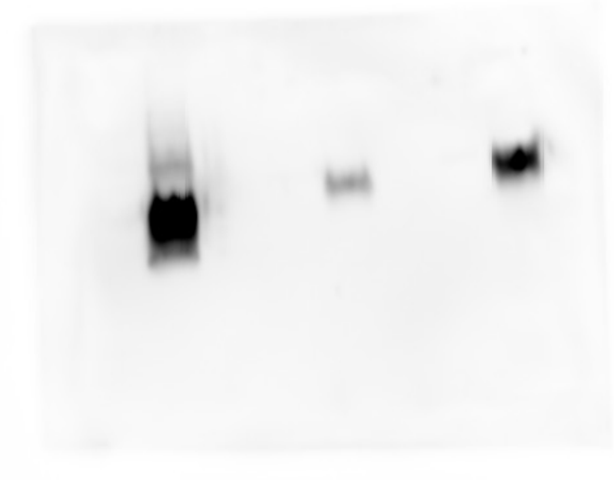

Lane 4 and 5/ probe of equivalent amount of the same protein samples in lane 4 and 5 shown in Figure S8E

J Raw Western blot data for Figure S10

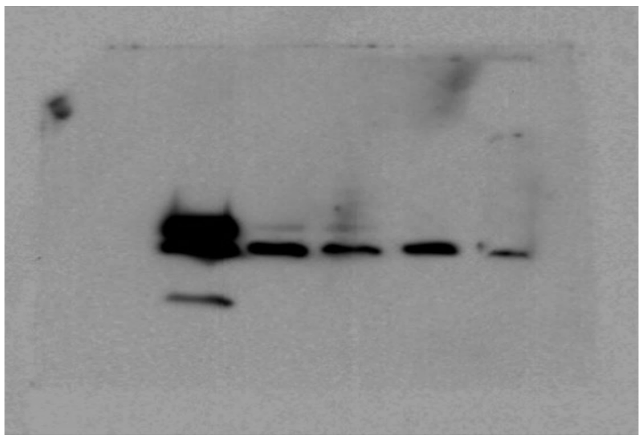

Lane 2 and 3/ probe of equivalent amount of the same protein samples in lane 2 and 3 shown in Figure S10B

K Raw Western blot data for Figure S10

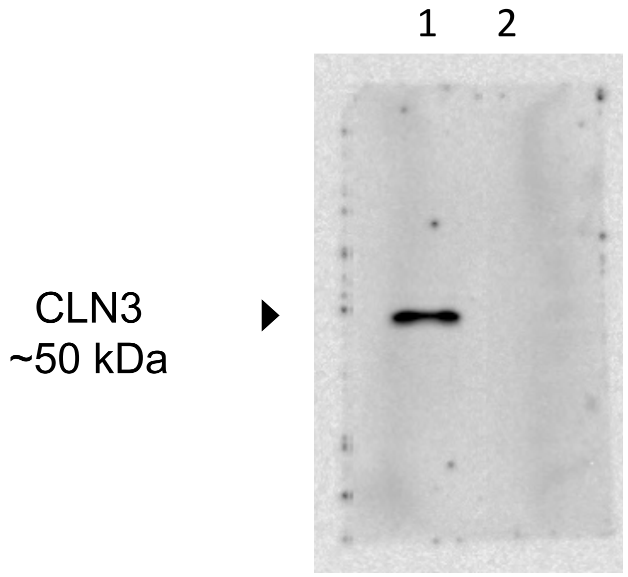

Lane 1 shown in Figure S10C

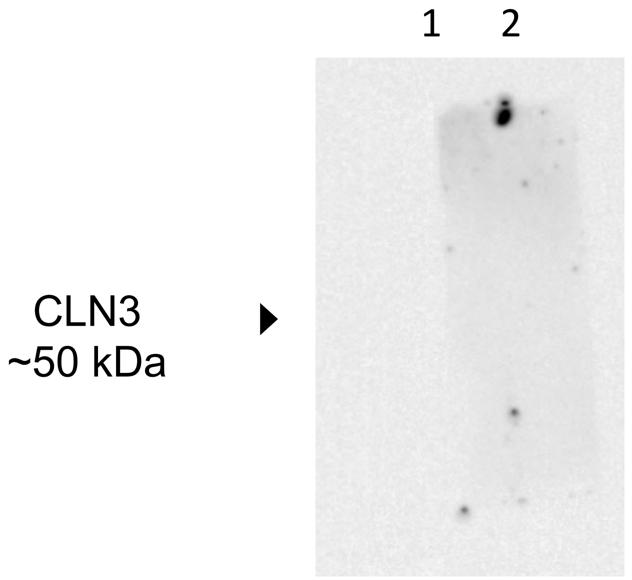

Lane 2 shown in Figure S10C

L Raw Western blot data for Figure S10

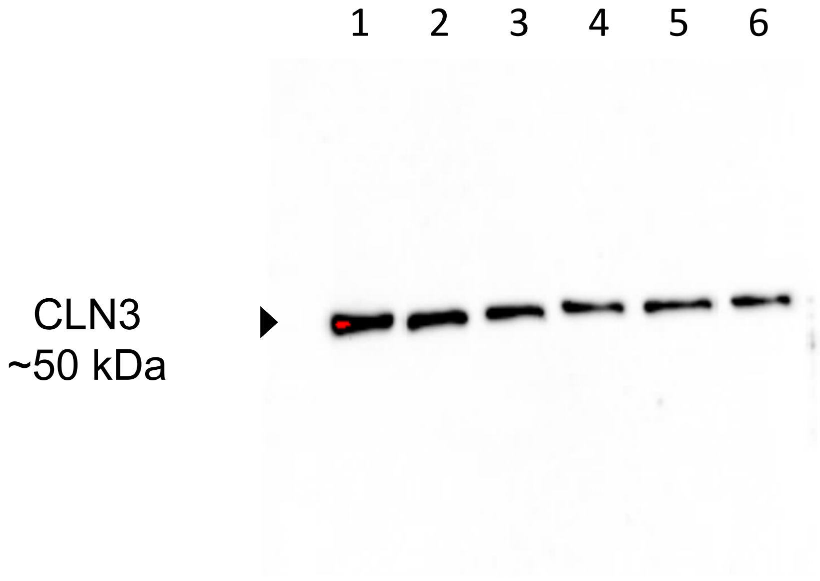

Lane 4 shown in Figure S10D

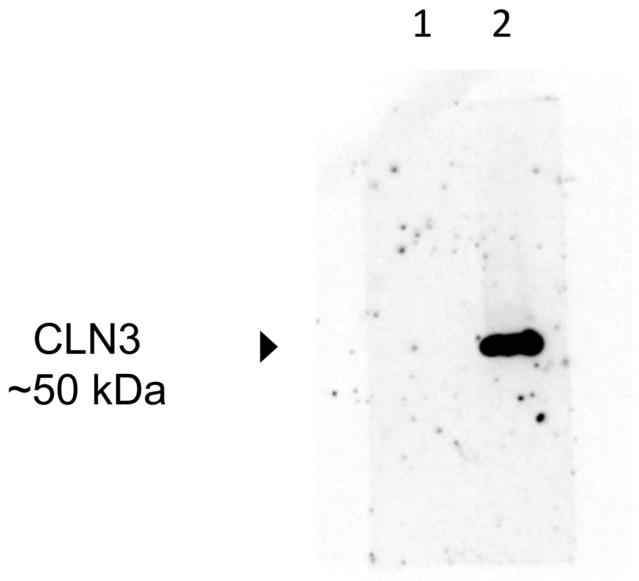

Lane 2 shown in Figure S10D

M Raw Western blot data for Figure S10

1 2

CLN3
~50 kDa

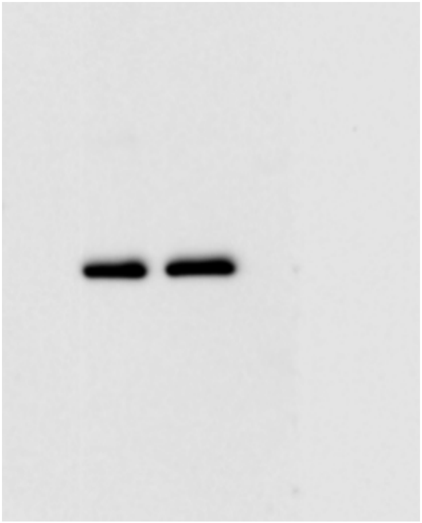

Lane 1 and 2/ probe of equivalent amount of the same protein samples in lane 1 and 2 shown in Figure S10F

1 2

EZR
81 kDa

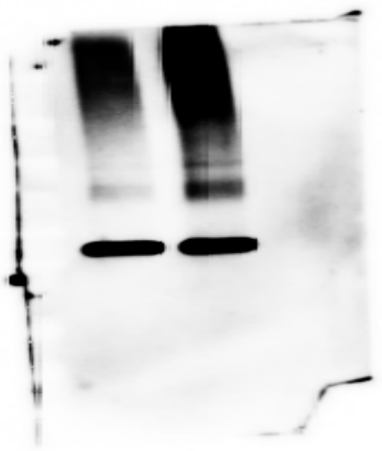

Supplementary Figure 13. Raw images showing Western blot data post image acquisition for all main and supplementary figures.

Supplementary Table 1:

1. *List of primers pairs (5'-3') used for quantitative real-time PCR analyses.*

Gene	Forward primer	Reverse primer
BEST1	*ATTTATAGGCTGGCCCTCACGGAA*	*TGTTCTGCCGGAGTCATAAAGCCT*
MERTK	*AGCCTGAGAGCATGAATGTCACCA*	*TGTTGATCTGCACTCCCTTGGACA*
MITF	*TTCACGAGCGTCCTGTATGCAGAT*	*TTGCAAAGCAGGATCCATCAAGCC*
PEDF	*AGATCTCAGCTGCAAGATTGCCCA*	*ATGAATGAACTCGGAGGTGAGGCT*
RPE65	*GCCCTCCTGCACAAGTTTGACTTT*	*AGTTGGTCTCTGTGCAAGCGTAGT*
OCCLUDIN	*TCCTATAAATCCACGCCGGTTCCT*	*AGGTGTCTCAAAGTTACCACCGCT*
CRALBP	*TTCCGCATGGTACCTGAAGAGGAA*	*ACTGCAGCCGGAAATTCACATAGC*
CLN3	*ATCAGGGCGCGCATTGGAA*	*ACAGCAGCCGTAGAGACAGA*
GAPDH	*AGCAAGAGCACAAGAGGAAGAG*	*GAGCACAGGGTACTTTATTGATGG*

2. Primer sequence used to detect the 1 kb deletion (to be included in the Methods section, paragraph hiPSC characterization

Forward: 5'- CATTCTGTCACCCTTAGAAGCC-3'
Reverse: 5'- GGCTATCAGAGTCCAGATTCCG-3'
