# Supplementary material for: A human model of Batten disease shows role of CLN3 in phagocytosis at the photoreceptor–RPE interface
Source: Commun Biol. 2021 Feb 5;4:161. doi: 10.1038/s42003-021-01682-5 (PMC7864947; doi:10.1038/s42003-021-01682-5)
Supplement: Supplementary file 3 — Description of Additional Supplementary Files [file 42003_2021_1682_MOESM3_ESM.pdf]

Description of Additional Supplementary Items

File Name: Supplementary Data 1

Description: Raw data for all main and supplementary figures
