# Supplementary material for: A human model of Batten disease shows role of CLN3 in phagocytosis at the photoreceptor–RPE interface
Source: Commun Biol. 2021 Feb 5;4:161. doi: 10.1038/s42003-021-01682-5 (PMC7864947; doi:10.1038/s42003-021-01682-5)
Supplement: Supplementary file 5 — Reporting Summary [file 42003_2021_1682_MOESM5_ESM.pdf]

nature research

Corresponding author(s): Ruchira Singh
Last updated by author(s): Nov 1, 2020

Reporting Summary

Nature Research wishes to improve the reproducibility of the work that we publish. This form provides structure for consistency and transparency in reporting. For further information on Nature Research policies, see Authors & Referees and the Editorial Policy Checklist .

Statistics

For all statistical analyses, confirm that the following items are present in the figure legend, table legend, main text, or Methods section.

n/a	Confirmed	
☐	☒	The exact sample size (*n*) for each experimental group/condition, given as a discrete number and unit of measurement
☐	☒	A statement on whether measurements were taken from distinct samples or whether the same sample was measured repeatedly
☐	☒	The statistical test(s) used AND whether they are one- or two-sided<br>*Only common tests should be described solely by name; describe more complex techniques in the Methods section.*
☒	☐	A description of all covariates tested
☒	☐	A description of any assumptions or corrections, such as tests of normality and adjustment for multiple comparisons
☐	☒	A full description of the statistical parameters including central tendency (e.g. means) or other basic estimates (e.g. regression coefficient) AND variation (e.g. standard deviation) or associated estimates of uncertainty (e.g. confidence intervals)
☐	☒	For null hypothesis testing, the test statistic (e.g. *F*, *t*, *r*) with confidence intervals, effect sizes, degrees of freedom and *P* value noted<br>*Give P values as exact values whenever suitable.*
☒	☐	For Bayesian analysis, information on the choice of priors and Markov chain Monte Carlo settings
☒	☐	For hierarchical and complex designs, identification of the appropriate level for tests and full reporting of outcomes
☒	☐	Estimates of effect sizes (e.g. Cohen's *d*, Pearson's *r*), indicating how they were calculated

Our web collection on statistics for biologists contains articles on many of the points above.

Software and code

Policy information about availability of computer code

Data collection	No software and code applied in the study.
Data analysis	No software and code applied in the study.

For manuscripts utilizing custom algorithms or software that are central to the research but not yet described in published literature, software must be made available to editors/reviewers. We strongly encourage code deposition in a community repository (e.g. GitHub). See the Nature Research guidelines for submitting code & software for further information.

Data

Policy information about availability of data

All manuscripts must include a data availability statement. This statement should provide the following information, where applicable:

- Accession codes, unique identifiers, or web links for publicly available datasets
- A list of figures that have associated raw data
- A description of any restrictions on data availability

Data availability statement: Source data for main and supplementary figures is provided in a supplementary data file. Inquiry of any additional data should be requested to the corresponding author.

Field-specific reporting

Please select the one below that is the best fit for your research. If you are not sure, read the appropriate sections before making your selection.

☒ Life sciences ☐ Behavioural & social sciences ☐ Ecological, evolutionary & environmental sciences

Life sciences study design

All studies must disclose on these points even when the disclosure is negative.

Sample size	All hiPSC-RPE experiments used up to three distinct control lines and always included two distinct CLN3 disease lines with n ≥ 3 for each individual experiment. In addition, two distinct clones per CLN3 disease hiPSC lines were utilized for experimental analysis. In each experiment, data from all control lines and CLN3 disease lines were grouped together and used in control vs. CLN3 disease analyses.
Data exclusions	Data was only excluded for failed experiments due to technical issues- for example antibody staining did not work and had to be repeated on the same sample
Replication	n ≥ 3 for each individual experiment from independent hiPSC differentiation runs. In addition, two distinct clones per CLN3 disease hiPSC lines were utilized for experimental analysis
Randomization	Data not randomized, as data compared control vs. CLN3 disease samples from human subjects and hIPSC-derived tissue
Blinding	Analysis of a subset of experiments, where experimenter bias could play a role (specifically analyses of electron microscopy data and quantification of bound vs. internalized POS and autofluorescent material accumulation in control vs. hiPSC-RPE) was carried out/confirmed in blinded analyses by investigators that did not perform data collection.

Reporting for specific materials, systems and methods

We require information from authors about some types of materials, experimental systems and methods used in many studies. Here, indicate whether each material, system or method listed is relevant to your study. If you are not sure if a list item applies to your research, read the appropriate section before selecting a response.

Materials & experimental systems

n/a	Involved in the study	
☐	☒	Antibodies
☐	☒	Eukaryotic cell lines
☒	☐	Palaeontology
☐	☒	Animals and other organisms
☐	☒	Human research participants
☒	☐	Clinical data

Methods

n/a	Involved in the study	
☒	☐	ChIP-seq
☒	☐	Flow cytometry
☒	☐	MRI-based neuroimaging

Antibodies

Antibodies used

BEST1 (Millipore) Cat# MAB5466
CRALBP (Abcam), Cat#ab15051
ITGA5 (Santa Cruz Biotechnology), Cat#sc-376199
ITGB5 (Abcam), Cat# ab184312
MERTK Abcam), Cat# ab52968
RHO (Millipore) Cat#MABN15
RPE65 (Millipore) Cat#MAB5428
CLN3, (Santa Cruz Biotechnology), Cat# sc-398192
CLN3 Blocking Peptide, (Santa Cruz Biotechnology), Cat# sc-398192P
EZR (Cell Signaling), Cat# 3145S
FLAG (Sigma), Cat# F3165
MYC (Cell Signaling), Cat# 2276S
GFP (Novus), Cat# NB600-308
OCT3/4 (Santa Cruz Biotechnology), Cat# sc-5279
NANOG (R&D systems), Cat# AF1997
LAMININ (Abcam), Cat# ab210956
LAMP1 (Abcam), Cat# ab24170
ZO-1 (Life Technologies), Cat# 61-7300
ACTIN (Santa Cruz Biotechnology) Cat# sc-47778

Alexa-Fluor 488 (Life Technologies), Cat# A21202
Alexa-Fluor 488 (Life Technologies), Cat# A21206
Alexa-Fluor 546 (Life Technologies) Cat# A10040
Alexa-Fluor 546 (Life Technologies) Cat# A10036

	Alexa-Fluor 633 Phalloidin (Life Technologies) Cat# A22284<br>HRP Secondary (Azure) goat anti-mouse, Cat# AC2115<br>HRP Secondary (Azure) goat anti-rabbit, Cat# AC2114
Validation	When applicable for WB, ICC and IHC analysis of primary antibody, by the manufacturer, prior publications in reputed journals and experiments in the laboratory to confirm expected tissue-specific localization in primary tissue and hiPSC-derived target cells. Of note, a subset of primary antibodies, that were essential for data validity, RHO and FLAG, analysis on used application (WB/ICC) was performed on positive vs. negative tissue/cell samples to confirm antibody specificity. Also, when applicable, the specificity of primary (CLN3) and secondary antibodies was similarly determined by evaluating specificity of staining in the presence and absence of primary antibody.

Eukaryotic cell lines

Policy information about cell lines

Cell line source(s)	293FT Thermo Fisher
Authentication	Used for lentivirus production and the viruses were transduced on human iPSC derived RPE cells
Mycoplasma contamination	Cell line not tested for mycoplasma contamination
Commonly misidentified lines<br>(See ICLAC register)	No misidentified cells were used in the study

Animals and other organisms

Policy information about studies involving animals; ARRIVE guidelines recommended for reporting animal research

Laboratory animals	laboratory animal tissue (mice) were only used to confirm CLN3 localization in RPE cells. Whole eyes from wild-type and CLN3-/- (Cln3Δex1-6) mice were from animals raised at Sanford research. All mice were cared for in accordance to animal protocols approved by Sanford Research's IACUC and in accordance with guidelines set forth by the NIH and AAALAC.
Wild animals	No wild animals were included in the study
Field-collected samples	No field-collected samples were included in the study
Ethics oversight	Sanford Research's IACUC

Note that full information on the approval of the study protocol must also be provided in the manuscript.

Human research participants

Policy information about studies involving human research participants

Population characteristics	Children aged 7 and 8 diagnosed with CLN3 disease
Recruitment	Not applicable--children diagnosed with CLN3 disease
Ethics oversight	University of Rochester Institutional Regulatory Board Protocol

Note that full information on the approval of the study protocol must also be provided in the manuscript.
